# Mortality in the L'Aquila (Central Italy) Earthquake of 6 April 2009

**DOI:** 10.1371/50585b8e6efd1

**Published:** 2013-01-07

**Authors:** David Alexander, Michele Magni

**Affiliations:** Institute for Risk and Disaster Management, University College London, London, United Kingdom; Università Politecnica delle Marche, Ancona, Italy

## Abstract

This paper presents the results of an analysis of data on mortality in the magnitude 6.3 earthquake that struck the central Italian city and province of L'Aquila during the night of 6 April 2009. The aim is to create a profile of the deaths in terms of age, gender, location, behaviour during the tremors, and other aspects. This could help predict the pattern of casualties and priorities for protection in future earthquakes. To establish a basis for analysis, the literature on seismic mortality is surveyed. The conclusions of previous studies are synthesised regarding patterns of mortality, entrapment, survival times, self-protective behaviour, gender and age. These factors are investigated for the data set covering the 308 fatalities in the L'Aquila earthquake, with help from interview data on behavioural factors obtained from 250 survivors. In this data set, there is a strong bias towards victimisation of young people, the elderly and women. Part of this can be explained by geographical factors regarding building performance: the rest of the explanation refers to the vulnerability of the elderly and the relationship between perception and action among female victims, who tend to be more fatalistic than men and thus did not abandon their homes between a major foreshock and the main shock of the earthquake, three hours later. In terms of casualties, earthquakes commonly discriminate against the elderly and women. Age and gender biases need further investigation and should be taken into account in seismic mitigation initiatives.

## Introduction

A magnitude 6.3 earthquake struck L'Aquila city and province in central Italy at 03:32 hours, Central European Time, on 6th April 2009. Some 308 people were killed at 19 different localities. In total, 1,500 people were injured, 202 of them seriously. About 100,000 buildings were severely damaged and 67,000 people were left homeless by the disaster. Data on the people who died, and the conditions under which they were killed are publically available**^1^** . They enable a detailed picture to be built up of the conditions at the time of death, the demographic characteristics of the victims and behavioural factors that may have influenced survival probabilities. Unfortunately, the same analysis cannot be applied to injured people, as, owing to loss of functionality of local medical centres, many of the wounded were dispersed to hospitals at diverse locations around central Italy and records of that process are not available. However, although the mortality data set is (thankfully) rather small, it gives an excellent opportunity to conduct research into the profiles and behaviour of victims in relation to the circumstances under which they died.

The magnitude M_W_=6.3 earthquake at L'Aquila (Figure 1) was a moderate seismic event. International researchers in this field have predominantly studied the largest earthquakes, such as the catastrophes that affected Haiti and Chile in 2010 and Japan in 2011. Moderate disasters are more common than these major events and deserve more attention by researchers than they have so far had. In the present case, the human effects were limited enough to enable detailed studies to be conducted at the level of individual victims.

**Location map.**
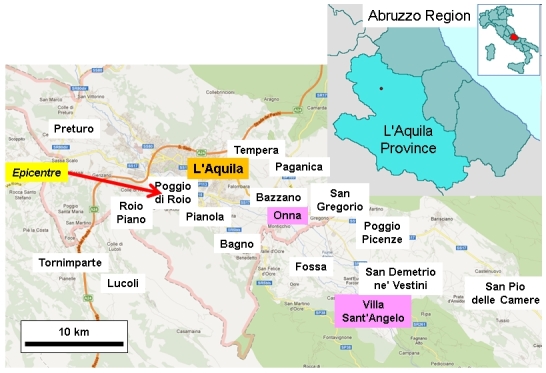
Image produced using maps from © OpenStreetMap contributors

Earthquake disasters are, above all, human tragedies in which loss of life and injury can be rapid or instantaneous and widespread. Mortality and morbidity in single events have been studied systematically since the 1960s**^2^**, but there is still much more to learn and characteristic patterns have not been fully established. If we are able to determine the common regularities in such events this will help emergency responses in the future by indicating what to expect and prepare for. It will also help reduce casualty totals by indicating the best ways to concentrate risk reduction initiatives.

This paper examines the patterns of mortality in the L'Aquila earthquake in simple demographic, spatial and behavioural terms, and in relation to the characteristics of buildings and damage. Other researchers have definitively established that the collapse of buildings is the principal cause of mortality in earthquakes (e.g. 3—among many others). The present work starts with the null hypothesis that, within the compass of this simple regularity, there was no difference of gender, age-group, occupation, building type or urban location in terms of the probability that a person would have died in the L'Aquila earthquake—i.e. that spatial, demographic, gender and social variations in the pattern of deaths were essentially random. This is abundantly not so, and the present article will establish what alternative hypotheses can be confirmed. In other words, it will seek the real determinants of mortality.

Before considering the data from L'Aquila, and in order to establish a basis for analysis, the next section will summarise the research on earthquake-related mortality.

## The Literature on Mortality in Earthquakes

Systematic study of mortality in earthquakes began in the 1960s and 1970s^3^ ^4^. Since then, most research has focussed on the outcome of individual seismic events (e.g. 3 on the Bam earthquake), but some researchers have aggregated data to produce a synoptic picture at the world scale (e.g. 5) or to create functions that link seismic damage with mortality (e.g. 6).

There is a strong consensus among researchers that building collapse is the main cause of death. As Albolghasemi et al. (3, p. 142) wrote in reference to the Bam earthquake of 2003 in Iran, "the main causes of morbidity were direct injuries, including fractures from collapse of structures, and the main causes of mortality were traumatic injuries and suffocation." This succinctly describes a typical pattern, which may nonetheless vary according to local circumstances.

In the literature on earthquake casualties, morbidity studies are more common than mortality research. This is because hospital admissions and treatment records furnish more data on which to base research. Moreover—very obviously—one can interview the injured but not the dead. However, owing to the effects of behavioural factors (i.e. self-protective actions), the profile of injuries may well be quite different to that of deaths. The following subsections will build up a systematic picture of what we know about earthquake-induced mortality.

**— The pattern of mortality**

Lomnitz^7^ analysed 21 Chilean earthquakes and found that the largest number of deaths occurred at night and in the home, a pattern replicated in many parts of the world (although not necessarily in North America, where the density and vulnerability of dwellings is commonly less than those of public buildings in city centres).

Despite the nocturnal factor, death and injury rates, and therefore the ratios between them, are highly variable from one earthquake to another^8^. Examples of typical mortality rates culled from the literature are as follows: 250 per 10,000 inhabitants (Armenia in 1988^9^); 46 (Egypt in 1992^10^); and 134 (Taiwan in 1999^11^). Reported injury rates were 2-150 times as large as death rates (at L'Aquila the figure was 4.87). With regard to individual settlements that had epicentral locations, death rates sometimes exceeded 10 per cent of the population^12^. Commonly, at least 85 per cent of the deaths occur within the first 24 hours after the earthquake, most of them instantaneously or in minutes. Instances of building collapse tend to decline rapidly with distance from epicentre: therefore, so do death rates^10^ ^13^.

The pattern of fatal injuries can also be highly variable. For example, the incidence of crush syndrome can vary from a handful of cases to about one third of victims 14. Moreover, heart attack can be a significant cause of death. According to Leor and Kloner^15^, the 1996 Northridge earthquake caused a 35 per cent increase in hospital admissions for acute myocardial infarction. Alexander^16^ found that about one per cent of victims in the 1980 southern Italian earthquake died from heart attacks (32 among 3,007 victims). The average age of the victims was 61.6 years and the range extended from 16 to 83 years. Allister^17^ described a case in which crush injuries caused life-threatening metabolic changes (hyperkalaemia and acidosis), that led to cardiac arrest, which indicated a link between crush syndrome and myocardial infarction. This highlights a serious problem of lack of standardisation of injury categories in the literature. Remedies for this have been proposed only very recently^18^

Nevertheless, in reference to the 1999 Taiwan earthquake, Liang et al.^13^ offered what might be regarded as a typical distribution of fatal injuries, in as much as such a thing exists: head injury 32.3%, traumatic shock 29.3%, and traumatic asphyxiation 29.1%. The second of these categories includes crush injuries.

**— Sources of death**

Anomalously, more than 80 per cent of deaths in the Loma Prieta (California) earthquake of 1989 occurred in motor vehicles, but the overall death toll was only 57^19^. More conventionally, Coburn and Spence^20^ found a good linear relationship between number of buildings collapsed and number of people killed, which enabled them to define a 'lethality ratio', representing the number of people killed per structure that collapsed. For the 1999 Taiwan earthquake, Liao et al.^21^ found that reinforced concrete (RC) construction was associated with a mortality rate six times lower than that of adobe and other forms of unreinforced masonry. However, when RC buildings collapsed, the death toll (or lethality ratio) tended to be higher. Liao et al.^21^ noted that housing performs much better if built to modern, rigorous building codes (in Taiwan these were introduced in 1974). Mahue-Giangreco et al.^22^ came to similar conclusions for the 1994 Northridge, California, earthquake. Excluding the Loma Prieta case, there is no doubt that building collapse is the main source of fatal injury. Moreover, after studying the 2002 Afyon earthquake in Turkey, Ellidokuz et al.^23^ found that being near an outside wall increased the risk of death 8.8 times with respect to partial collapse, but being in a house that collapsed completely increased it more than 70 times.

**— Entrapment and survival time**

Macintyre et al.^24^ and Safar^25^ argued that in major earthquakes many lives could be saved by better, more prompt urban search and rescue (USAR). Referring to the Bam (Iran) earthquake of 2003, Mirashemi et al.^26^ showed experimentally that being trapped under rubble for as long as six hours significantly reduces the probability of survival, while Naghi et al.^27^ showed that delays in treating crush injuries substantially increase the chances of renal failure. In a rather extreme case, DeBruycker et al.^28^ noted that in the southern Italian earthquake of 1980, 95% of deaths occurred among trapped people who died before they could be rescued. In contrast to these examples, in the L'Aquila case prolonged entrapment was not a major determinant of mortality.

**— Behaviour in earthquakes**

One of the least well understood aspects of casualties in earthquakes is the role of self-protective (or, conversely, risky) behaviour. This is not a simple matter. Armenian et al. (29 p. 254) noted that: "Confusion about recommended first actions might arise because the relative efficiency of protective occupant actions is very much dependent on the seismic performance of specific building types." Archea and Kobayashi^30^ observed that Japanese behaviour in earthquakes is generally maladaptive. In contrast, Goltz et al.^31^ found that in California “behaviour in a rapid onset disaster is controlled, rational and adaptive.” Whether or not that betokens a cultural difference with implications for saving lives demands further investigation (see 32).

A common tendency among people in buildings is to rush outside. According to DeBruycker et al.^33^, 55 per cent of survivors of the 1980 southern Italian earthquake did so. It is not known how many of those who were fatally injured tried to run outside in southern Italy, but in the 1976 Friuli (NE Italy) earthquake, Hogg^34^ found that agile people were most at risk of death, because they rushed out and were crushed in the street by falling masonry.

**— Gender issues**

The seminal paper on gender differences and discrimination in disasters was published in 1982 by Rivers, who observed (35 p. 257) that "Sex differences in deaths or injuries of event victims can be expected to occur where sex differences in social role result in exposure to different environments." He was referring in part to Beinin's^36^ observation that in the nocturnal Ashkabad (Russia) earthquake of 1948, almost two and a half times as many women as men died, while in the 1966 Tashkent disaster five women died for every four men. Beinin thought that women burdened by children found it more difficult to escape. In other words, they sacrificed themselves for their offspring and their husbands did not do so. This is possible, but it remains contentious in other cases, including the one analysed here (see below).

One of the worst cases of gender discrimination described in the literature is that of a sewing factory which collapsed in the 1988 Armenian earthquake, with 97 per cent mortality among the 212 occupants, the majority of whom were women^37^. Other authors have found a predominance of women in the overall death tolls, for example in the 1990 Manila, Philippines, earthquake (55 per cent^38^), Northridge, California, 1994^39^, Taiwan 1999^11^ and Pakistan, 2005^40^. In each case, the gender bias existed in both mortality and morbidity statistics. For example, in Taiwan, psychiatric morbidity was more prevalent in women than in men (41—this was also true in L'Aquila: 42). Cases in which male mortality predominates over female are rare and seem to be restricted to earthquakes with low mortalities, as in Loma Prieta, California, 1989^19^, and Athens, 1999^43^. On the contrary, Baba et al.^44^ found that females constituted 59 per cent of 5,502 deaths registered as a direct consequence of the 1995 Kobe earthquake in Japan.

In a study of the aftermath of the 1999 Taiwan earthquake, Kung and Chen^45^ found that women had a heightened perception and greater fear of earthquakes, especially if they also had low educational achievement. In the Kashmir (Pakistan) earthquake of 2005, Hamilton and Halvorson^46^ found that women were substantially more at risk than men, largely because behavioural factors interacted with the vulnerability to collapse of poorly-built dwellings. The earthquake occurred when more women were indoors than men^40^. Purdah trapped women inside their homes and imposed a cultural barrier that stopped them from running outside (and in fact where Purdah was less of a problem, a greater proportion of women survived). After the main shock women suffered greater deprivation and hardship than men but did more to pull families out of the crisis situation. Working in Peru, Shenk et al.^47^ discovered that males tended to focus on the tangible effects of the disaster, while females addressed emotional concerns and spent more of their energies on domestic affairs and church worship. The same appears to have been true in L'Aquila.

A classic epidemiological study of casualties is that by Glass et al.^48^ of the Guatemala 1976 earthquake. This event occurred at 03:05 hrs and caught people at home. It killed 22,778 people and injured 76,504. There was relatively high mortality among children in adobe houses. Given the emphasis on age groups, it is astonishing that Glass and his colleagues paid almost no attention to gender.

If women are indeed more likely to die in earthquakes than men, the urgent question is why? Petal^49^ assumed that the answer is necessarily behavioural, but despite the work of authors such as Hamilton and Halvorson^46^, evidence from the literature is in very short supply. Gender information is conspicuous by its absence; for example, in Nichols's and Beavers's^6^ world earthquake fatality function, and various casualty prediction models (e.g. 50 51). Moreover, in the 1999 Kocaeli earthquake in Turkey, Ramirez et al.^52^ found that girls were twice as likely to become casualties as boys. In studies such as this, no explanation was offered for the gender difference.

**— The elderly**

If the research mentioned in the previous subsections reveals a catalogue of partial and unexplained regularities, there is much more certainty about the vulnerability of the elderly to harm in earthquakes^47^. In the 1994 Kobe disaster, more than half of the people who died were older than 60, and yet they made up only 17.8% of the population^53^. In this case, many pensioners lived on the ground floor and were the victims of soft-storey collapse. Not only are older people more frail, less mobile and in some cases less perceptive than younger people, pensioners in places like Kobe tend to have inexpensive homes that are less resistant to earthquake damage. In Northridge, 1994, the elderly were almost three times as likely to be injured as were young adults^39^. In the 1999 Taiwan earthquake, mortality among people over 80 years old was an order of magnitude higher than that of people in their twenties^13^.

It is remarkable that very few of the studies of mortality by age-group examined gender biases, and those that did made little or no attempt to make corrections for gender imbalances in the population in which the deaths occurred (for example, by taking into account the fact that commonly more women than men are alive in old age). Moreover, explanations of the patterns found tended to be vague or even completely lacking.

**— The emerging picture**

Mortality in earthquakes tends to be dominated by nocturnal events that catch people while they are sleeping, or at least while they are inside buildings that are vulnerable to instantaneous collapse. Patterns and rates of death can be highly variable from one event to another (and so can distributions of types of injury), but there are some regularities. The elderly are most at risk, and in some events that is also true of small children, although in other cases infants and toddlers are readily saved by their parents. Hence, there is little opportunity to make a firm conclusion about whether specific behaviour patterns in earthquakes save or sacrifice lives.

Crush injuries account for a variable but often substantial proportion of fatal and non-fatal pathologies^54^. The statistical weight of these and other injury types in mortality patterns is mediated by entrapment and time to rescue, which can significantly reduce the chances of survival.

There is strong but sporadic evidence in the literature that earthquake mortality involves a gender bias, with women more at risk than men. Available published studies are distinctly disappointing, in that there is a pervasive tendency not to follow up this issue and or seek explanations. The remainder of this paper will attempt to redress the balance on this and other outstanding issues. To begin with, the next section will offer a description of mortality in the L'Aquila earthquake.

## Patterns of Mortality in the April 2009 L'Aquila Earthquake

A remarkable and perhaps unique data set of precise information on the people who died in the L'Aquila earthquake was assembled by the local newspaper,* Il Centro*, and is in the public domain at http://racconta.kataweb.it/terremotoabruzzo/. We used the information it contained and supplemented it by field investigation of virtually all the sites concerned.

The death toll in the earthquake was dominated by L'Aquila city (2009 pop. 63,688), not least because it was a reservoir of population 24 times bigger than the next largest affected settlement (Tornimparte, 3,002 inhabitants). L'Aquila is a very extensive municipality (467 km^2^, pop. 72,988) that contains 14 villages and 7 hamlets. Many of these experienced no deaths, although eight of them produced a total of 65 fatalities. The satellite communities within the municipality vary in population size from a few hundred inhabitants to, in the case of Paganica (five deaths), more than 5,000 (Table 1, Figure 2).

**Table 1. Deaths and population in the L'Aquila area.** 

Settlement	Comune	Deaths	Population	Males	Females	Deaths/ popn.
L'Aquila	L'Aquila	202	72,988	35,077	37,911	0.32
Onna	L'Aquila	39	[310]	--	--	12.58
Villa Sant'Angelo	Villa Sant'Angelo	17	433	206	227	3.97
San Gregorio	L'Aquila	8	[433]	--	--	1.85
Tempera	L'Aquila	8	[900]	--	--	0.89
Paganica	L'Aquila	5	[5,024]	--	--	0.10
Poggio Picenze	Poggio Picenze	5	1,066	513	553	0.47
San Pio delle Camere	San Pio	5	622	317	305	0.80
Fossa	Fossa	4	701	343	358	0.57
San Demetrio ne' Vestini	San Demetrio	3	1,834	923	911	0.16
Pianola	L'Aquila	2	--	--	--	--
Roio Piano	L'Aquila	2	[500]	--	--	0.40
Tornimparte	Tornimparte	2	3,002	1,481	1,521	0.07
Bazzano	L'Aquila	1	[600]	--	--	0.17
Civita di Bagno	L'Aquila	1	--	--	--	--
Lucoli	Lucoli	1	1,012	510	502	0.10
Poggio di Roio	L'Aquila	1	[733]	--	--	0.14
Preturo	L'Aquila	1	[800]	--	--	0.13
Sant'Angelo di Bagno	L'Aquila	1	--	--	--	--
Totals	--	308	81,658	39,370	42,288	0.37
NB: Figures in square brackets are population estimates for settlements that are not full municipalities.						

Considered on logarithmic scales, the relative death toll appears to decline with increasing population size until a break-even point of about 2,500 inhabitants, and then it increases (Figure 3). With such a small data set (thankfully a relatively small number of deaths), it is difficult to verify this statistically, but it may represent two processes. Regarding the first of these, there were some particularly vulnerable villages, such as Onna (population 310), in which 39 people died. In this locality, anomalously high accelerations on soft sediments led to the devastation of about 60 per cent of the building stock. Other small settlements (e.g. Villa Sant'Angelo: 433 inhabitants, 17 deaths) suffered from the poor quality and high vulnerability of their historical unreinforced masonry building stock. Under the second process, the large settlements offered more varied and numerous cases of vulnerable urban fabric and higher population densities in the places that were most at risk.

**Location of deaths, by town.**
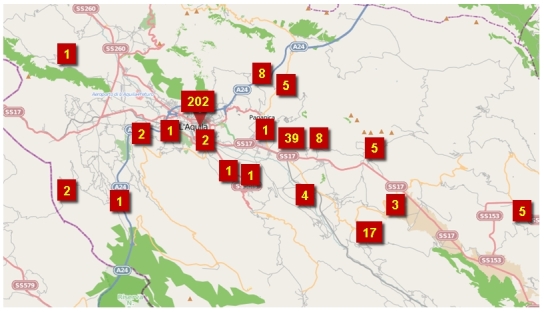
Image produced using maps from © OpenStreetMap contributors

**Death tolls in relation to population size of settlements.**
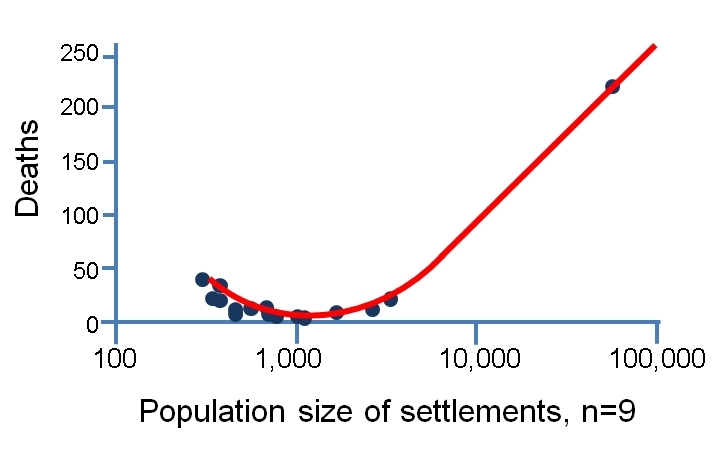

Some 93 per cent of the 308 victims were Italians by birth. More than half were natives of the affected towns and villages and 65 per cent were from L'Aquila Province (Figure 4). Many of the others had long-term connections with L'Aquila and very few were entirely extraneous, as the area does not have mass tourism and, in any case, the earthquake did not occur during the tourist season. It therefore appeared reasonable to use the demographic profile of L'Aquila Province in 2009 as a point of reference for the analysis of the age-sex profile of victims. It should be stressed that there is nothing visibly anomalous about this with respect to the other provinces of central Italy, excepting, perhaps, Rome. Only four victims (1%) were from Rome. The average age of victims was 51.1 years. The youngest was aged only a few months and the oldest where a man and a woman (unrelated to each other) who were both 96.

**Birthplaces of people who died in the L'Aquila earthquake.**
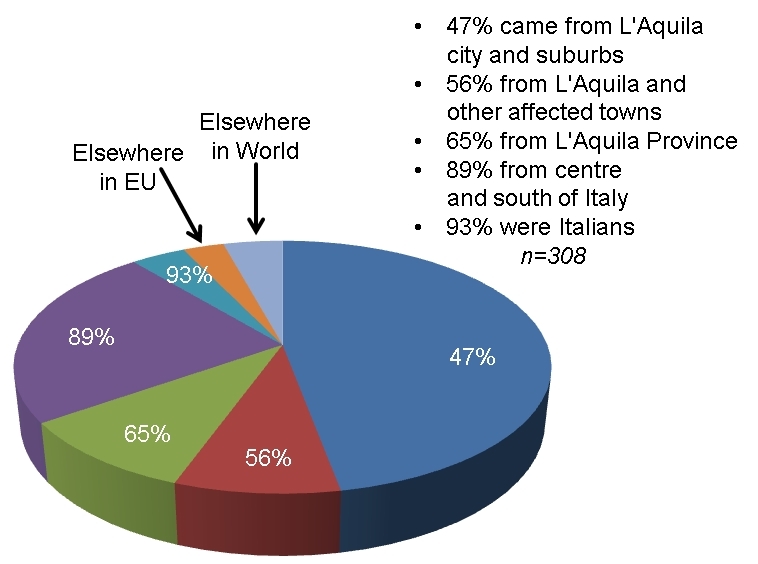

In order to analyse the discrepancy between demographic profiles and the actual age-sex distribution of deaths, three models were used. In the first, the 308 deaths were apportioned to each age and sex class according to the relative population size. In the second, a constant rate of 7 deaths per 10,000 of the population was used, which was derived from the death toll in relation to the total population of L'Aquila Province. In the third model, a constant rate of 38 deaths per 10,000 of the population was employed which was derived from the death toll in relation to the total population of the eight municipalities and 19 settlements that experienced deaths in the earthquake. The only differences found between the results of the models were in the degree to which they accentuated the discrepancies between a (purely theoretical) distribution of deaths based on the demographic profile of L'Aquila and the actual distribution. They all showed the same pattern (e.g. Figure 5).

The null hypothesis is that there should be no difference between the distribution of deaths by age and gender and the demographic profile of the population. This is easily disproved. The first major anomaly is in the high number of deaths in the 20-29 years age-group. These amount to between 27 and 40 excess fatalities (8.0-13.0%), according to which of the three models is used. This reflects the presence of numerous students in L'Aquila and the relatively low seismic resistance of the accommodation in which they lived. For instance, in one notorious case, 11 people died in the instantaneous, total collapse of one wing of the *Casa dello Studente*student dormitory, a multi-storey reinforced concrete building. The numbers of males and females who died in this age group are approximately equal.

**Anomalies in mortality rates by age and gender, L'Aquila earthquake.**
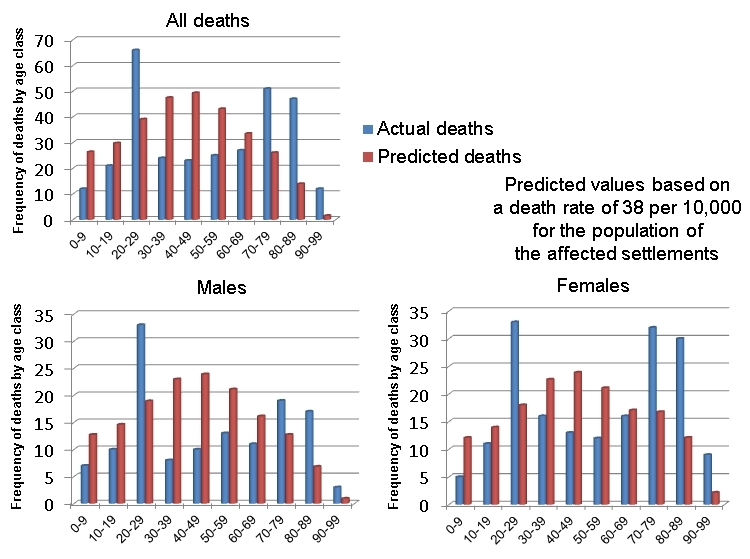

A somewhat larger anomaly is represented by deaths among people over the age of 70. This is in the range 59-74 people (19.2-24.0%). For all three models, the anomalies are about twice as large for women as for men. This indicates that the earthquake caused a disproportionate number of victims among old people, but with a very high bias towards old women. This cannot be explained alone by the fact that, in 2009 in L'Aquila Province, the predominance of women over men in the 70-100 age groups was 52.6 per cent, amounting to 10,670 individuals. Overall, 42.5% of the dead were men and 57.5% were women, a substantial difference and one that corresponds very well with earthquakes elsewhere (e.g. 38 ,44). Death rate anomalies are depicted for the three models in Figure 6.

The positive anomalies consist of an unexpectedly low death toll among infants, children and mature adults in the 30-70 age groups. However, there is one further negative anomaly in the death tolls: twice as many women were killed as men in the 30-39 age-group. The numbers were not large (8 and 16, respectively), but the difference is thought provoking. This aspect is dealt with more extensively below.

**Death rate anomalies for age and gender (three models).**
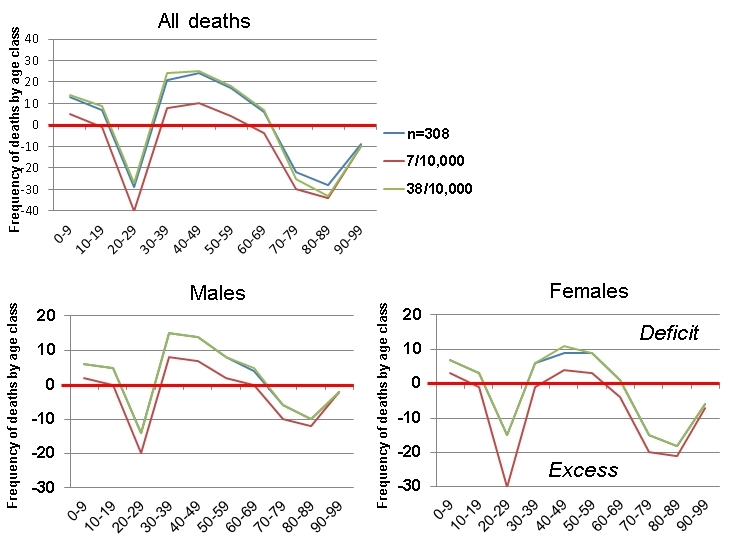

To recapitulate, there is a need to explain, or clarify, the following anomalies in the demographic distribution of deaths in the L'Aquila earthquake:-

- unexpectedly low numbers of infants and children died
- high numbers of young adults were killed
- twice as many women as men in their thirties died
- people over the age of 70 were heavily victimised: death tolls among women in this age group that was twice as large as those among men, despite correcting for the relative balance of people alive in old age.

The answers to the questions raised by this analysis are not easy to find. In part this is because the sample size (n=308) is small. In addition, many potential explanations are not well supported by evidence. For instance, one hypothesis that seemed worth testing is that the average age of victims is higher in the outlying villages than in the central city and its immediate environs. The reason for this would be that small and relatively isolated villages tend to be populated by retirees, as they are not near places of work, education and social activities. Unfortunately, the evidence is too vague to confirm the hypothesis (Figure 7), and the large differences in death tolls between l'Aquila and the villages rule out statistical analysis.

**Average age of victims, by settlement.**
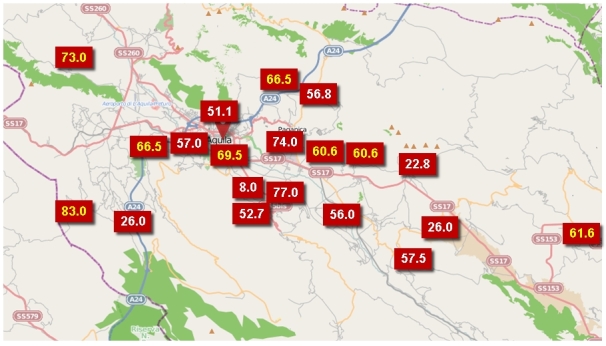
Image produced using maps from © OpenStreetMap contributors

The data set of mortality in the L'Aquila earthquake includes, not only the demographic information on the victims (age, sex, birthplace, etc.), but for 90.3 per cent of cases the location of the building in which they died is known either exactly (56.2% of cases) or with a very good approximation (34.1%). This factor can be compared to the pattern of building collapses. In many cases, exactly what happened to the victim during the earthquake is recorded in the data set. This enabled us to determine that there were six cases of heart attack (2% of the victims). In at least four of these cases, the heart attack was associated with injury due to structural collapse. There were also 16 cases of delayed death in hospital (over intervals that varied from several hours to 12 days) and one during pre-hospital care. These cases account for 5.5% of the victims. One cause of death in hospital was pneumonia and several were related to shock. The cause of death was unknown in 18 instances (5.8% of all cases). In 260 cases (87%), death was instantaneous or rapid and was caused by blunt trauma, crush injuries or asphyxiation as a result of the fall of a load-bearing structure directly onto the victim. This corresponds to the findings of previous studies (see above). Hence, the vast majority of deaths occurred rapidly or instantaneously when the buildings in which the victims were located collapsed.

Fifteen of the victims were found under the rubble clasping a family member for support or to shield them, and seven were evidently trying to save familiar or valuable objects, which they had in their hands. It is, of course, impossible to gain a clear, comprehensive picture of the state of mind of the victims at the time of the earthquake. They cannot be interviewed, and 70% of them were not in the presence of anyone who survived and could reconstruct the situation afterwards. However, it is known that at least 25 of the victims were very worried about the risk of an imminent earthquake. This led some to sleep on a couch near the main exit to the house, and others to telephone family members repeatedly. Nonetheless, there is evidence that another 34 of the victims were calm and fatalistic enough to have come to terms with the violent foreshock that occurred three hours before the main shock. Some were evidently asleep when crushed by falling masonry.

Although it is not possible to know exactly what all the victims were doing at the exact moment of the earthquake, it is known that at least 44 of them were in bed, having returned there after the foreshock which occurred at 00:30 hrs. Most of the others were in bedrooms and so were more than three quarters of the interviewed survivors (see below).

These observations help to build up a partial picture of how and why people died, but they offer little insight into the anomalies described above. It should be borne in mind that the main shock occurred at 03:32 hrs in the morning, and hence some behavioural explanations for the gender anomalies are invalidated by the fact that men and women were not likely to be engaged in particularly different activities at that time of the night. However, although the foreshock that occurred three hours before the main shock did not do any damage, it caused much alarm and induced very many people to leave their homes. They were then faced with the choice of whether to go back again or go away. We conducted a systematic programme of interviews with survivors (see next section), and this tended to suggest that women were sometimes more fatalistic than men about what the foreshock would lead to, or that they had more of a tendency to regard the home as a refuge and place of safety.

As a step towards understanding what the perception of people who died might have been, we will now consider how people who survived reacted to the earthquake.

## A Survey of L'Aquila Survivors

The second author of this paper interviewed 250 survivors in the L'Aquila area, during the course of a broader survey of the health and welfare of survivors lodged in transitional housing provided by the Italian national and regional governments. The sample has an approximately equal division between males (52%) and females (48%). All of them had been made temporarily or permanently homeless by the earthquake, which destroyed or seriously damaged 100,000 buildings, including the homes of 67,000 residents. With respect to the local demographic profile, the sample involved some under-representation of children and teenagers and of people over the age of 80. It thus over-represented people between 40 and 79. This reflects an embargo on interviewing children and difficulties of finding and speaking with very aged respondents, as well as the need to work with whoever could be contacted and was willing to take part in a lengthy questionnaire survey.

Virtually all interviewees were indoors when the earthquake struck. In answer to the question "What did you do during the main shock?" almost one in seven people replied that they had sought refuge in their houses. Slightly more than one in three tried to escape from home and a similar proportion did nothing (i.e., their actions were passive). Thus, slightly more than half engaged in active behaviour.

The results of two-tailed t-tests show a significant difference between the mean age of people who tried to rush outside during the earthquake (50.9 years) and those who sought refuge at home (33.5 years); t(106)=3.935, p=0.000 (two-tailed). A similar, also significant, difference was found between the ages of people who engaged in passive behaviour (51.9) and those whose behaviour was active (33.5 yrs); t(107)=4.28, p=0.000 (two-tailed). Hence, among the survivors in the sample, the incident of active behaviour decreased with age and that of passive behaviour increased correspondingly (Figure 8). Paradoxically, the tendency to seek refuge at home diminished with age, even though the tendency to flee the building increased. At the time of the earthquake (03:32 hrs), 77 per cent of respondents were in their bedrooms. We can conclude that with increasing age the dilemma of what to do tended to resolve itself into two options: try to get out or do nothing.

**Behaviour during the earthquake of interviewed survivors.**
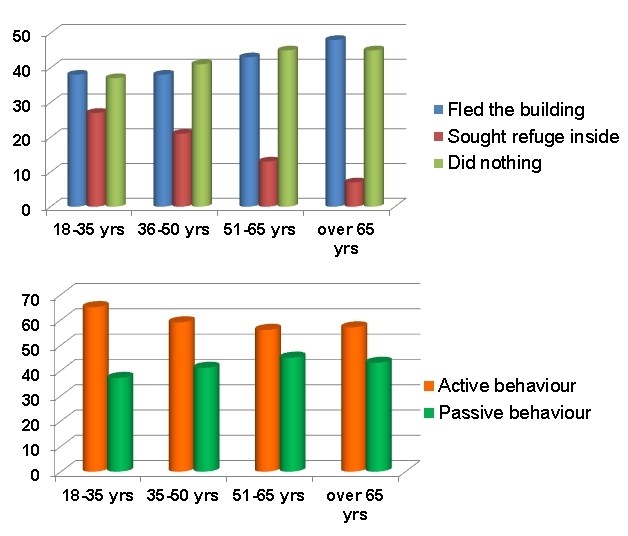

Active behaviour was more common in people who had a higher level of educational achievement, than among those who had only completed basic school education (there was a 14% difference, whose significance is confirmed by a Chi-square test). There was no significant difference between people who engaged in active or passive behaviour during the earthquake in relation to the type of building in which they were located (unreinforced masonry or reinforced concrete). Significantly higher levels of active behaviour were encountered in people who lived in condominiums in relation to those who lived in single dwellings.

Next, an analysis was conducted for the female population of interviewees. An independent T-test was performed to compare the average age of female respondents with the behavioral response to the mainshock. The 'behaviour' variable encompassed three groups: (1) people who sought shelter, (2) those who immediately tried to escape from inside buildings and (3) those who did not move during the earthquake. A statistically significant difference (t (77)= -3.21 ,p<0.005) was found between group 1 (M = 32, SE = 2.35) and group 2 (M = 42, SE = 2.14) and the magnitude of the differences in the means showed a medium-sized effect (r = 0.34). A meaningful difference (t (85)= -4.321, p<0,005) with a medium-sized effect (r = 0.42) was also observed comparing group 1 (M = 32, SE = 2.35) and group 3 (M = 47, SE = 2.3). There was no significant difference (t (122)= -1.632, p=0.1) in age for group 2 (M = 42, SE = 2.14) and group 3 (M = 47, SE = 2.3) and this constituted a small-sized effect (r = 0.15).

A further independent T-test was conducted after merging groups 1 and 2 into a single group consisting of respondents who engaged in an active response to the tremors, while group 3 consisted of those whose response was passive. The analysis reported a significant difference (t (153)= -2.92, p<0.005) in the means for people who behaved actively (M = 39, SE = 1.71) and passively (M = 47, SE = 2.3) with a small-sized effect of r = 0.23. Furthermore, whether there was a pattern in the medians of age for groups 1, 2 and 3 was investigated. A Jonckheere test^55^ was used, which revealed a significant trend in the median values of age across the groups (J = 5018.5, z = 3.99, p<0.001). Therefore, the medians increase moving from the first group (24±13.09 ) through the second (39±17.67) to the last one (48.5±17.19).

The same tests and analysis applied to the all-male group of interviewees revealed no statistical difference between different groups. From this we can conclude as follows:-

- the behaviour of males in the sample was heterogeneous and not characterised by a pattern, while that of females was more easily differentiated, as follows;
- in females the incidence of passive behaviour increased with age;
- the youngest females tended to seek shelter, while the oldest did nothing.

In synthesis, in terms of the first reaction to the earthquake, active behaviour prevailed over passive reactions, and tendency to flee the building increased with age, as did the tendency not to react in the heat of the moment. The behaviour of men was not differentiated by age but that of women was.

If we relate the results from the sample of living survivors to that of the population of dead victims, the implication is that the tendency to do nothing could prove fatal in an earthquake such as that which occurred at L'Aquila. Although running outside is not necessarily a safe option, doing nothing is potentially even less safe. This implies that in seismic disasters self-protective behaviour does have a role and that women, especially elderly women, need to be encouraged or helped to adopt it.

## Patterns of Mortality in L'Aquila City

As noted, L'Aquila city was, predictably, the major source of mortality in the 6 April 2009 seismic disaster. Maps of the location of deaths (Figures 9-11) were generated by plotting the available data on topographic base maps and Google Earth images of the city and verifying their accuracy by field investigation amid the damaged buildings.

Sixteen deaths (7.9%) occurred in dispersed locations outside the city centre, while the other 186 (92.1%, or 60.4% of the total mortality in the earthquake) were highly concentrated in central locations. Despite all that has been written about the relative safety of reinforced concrete frame (RC) buildings in comparison with unreinforced masonry (URMB) ones, 39 deaths (21.0%) occurred in the area of unreinforced masonry buildings and 147 (79%) in areas of newer RC buildings. To understand this situation it needs to be borne in mind that in L'Aquila city centre there were four areas of different seismic performance (Figure 9), and thus different patterns of mortality^56^:-

(a) In the northeast quadrant, buildings (predominantly URMBs constructed in diverse periods over the last 300 years) were well maintained and there were no deaths. No particular seismic amplification, soil or foundation factors were at work here. Nevertheless, the area contains L'Aquila's 16th century castle, which was badly damaged.

(b) In the northwest quadrant casualties were distributed thinly across a wide area and were mainly caused by roof and stairway collapse, as well as masonry that cascaded into the streets. The area is largely composed of URMBs of diverse ages, including buildings many hundreds of years old.

(c) In the southeast quadrant there was limited damage were few casualties and the situation was essentially mid-way between that of the northeast and northwest quadrants. The building stock here was dominated by URMBs of diverse ages.

(d) Along the southwest fascia of the city topographic amplification occurred, as well as consolidation subsidence. The area is dominated by RC buildings constructed before the latest seismic codes were introduced (especially in the 1960s and 1970s) and with frames that lacked adequate stiffness to resist deformation. One quarter of casualties in L'Aquila city occurred in the collapse of only seven RC buildings, in each of which there were was a death toll of between 6 and 25 people. Almost all cases of multiple deaths (more than two at one location) occurred in this area.

**Zones of building collapse in L'Aquila city centre.**
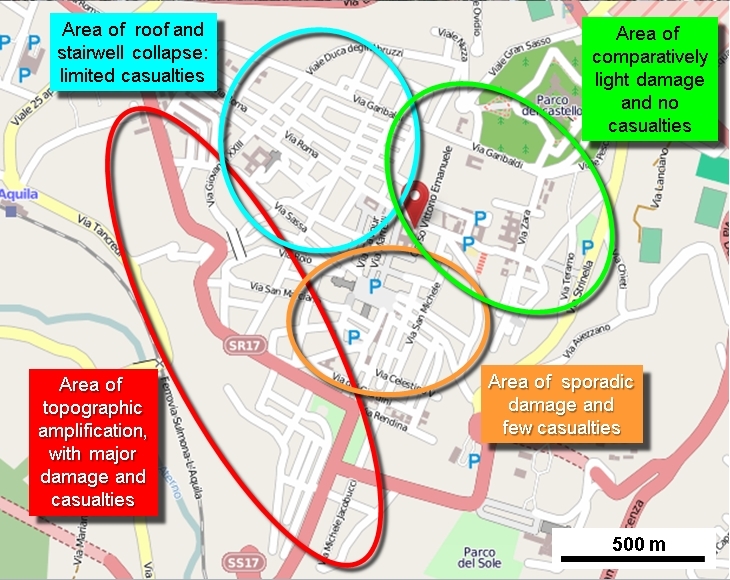
Image produced using maps from © OpenStreetMap contributors

**Locations of deaths in L'Aquila city centre, by building type-zone.**
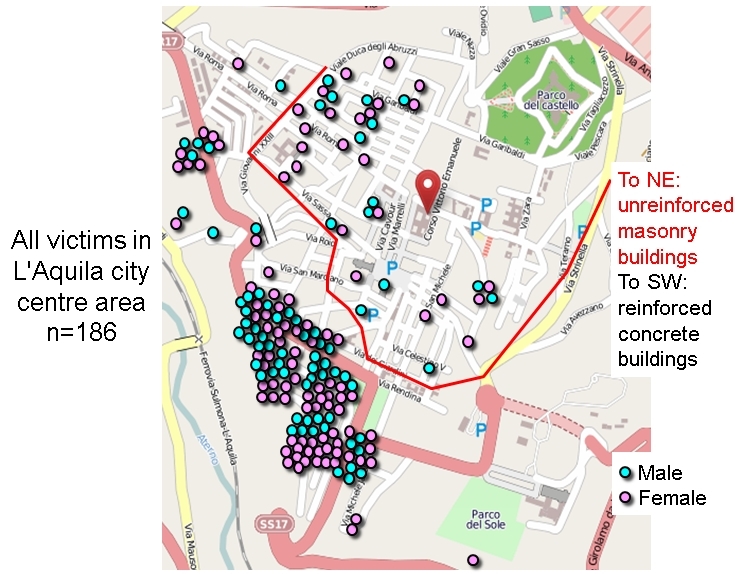
Image produced using maps from © OpenStreetMap contributors

Hence four out of five casualties occurred in an area outside the main core of historic buildings (Figure 10). It is notable, however, that there is a distinct spatial clustering of victims of certain ages. Elderly victims (70-96 years) were found predominantly in the northwest quarter and young victims (18-29 years) were grouped in the southwest area. This, of course, reflects the clustering of different kinds of residence (Figure 11).

The victimisation of elderly people in earthquakes has been repeatedly demonstrated in mortality statistics^13^ ^57^ and is amply confirmed by this study. In the L'Aquila data set, above the age of 70 there are 19-24% more deaths than predicted by local demographic profiles, amounting to 59-74 individuals out of 308. In L'Aquila Province, 16.5% of the population is over the age of 69. If the 308 deaths were proportional to the demographic profile, there would have been 33 in L'Aquila city, six or seven in Onna and three in Villa Sant'Angelo. In reality there were respectively 66 in L'Aquila city (although only one third of them in central city, which was given over to student accommodation, families and commercial premises), 16 in Onna and six in Villa Sant'Angelo. These numbers are at least double those expected under the null hypothesis. As the cause of death is known (in more or less detail) from descriptions of specific circumstances in 94.2% of cases, it is evident that the frailty and lack of mobility of old people put them at risk. This tends to contradict Hogg's (1981) finding that agile people were most at risk: so does the substantial positive anomaly among adults in the 30-69 age groups, amounting to between 18 and 74 lives not sacrificed, depending which model is used. However, it is as well to bear in mind that the sample size is small and some anomalies could conceivably be an artefact of that. Nonetheless, a second factor may be that in many Italian towns elderly pensioners tend to live in less well-maintained accommodation, and especially in vulnerable historic buildings that have been family homes for centuries (cf. 53).

A very substantial negative anomaly is represented by the high mortality among individuals in the age-group 20-29 years (Figure 5). It amounted to between 27 and 40 people, depending on which model is used. This is unusual, but not unique. For example, in the 1990 Manila, Philippines, earthquake (1,084 deaths), educational facilities collapsed in two towns, causing heavy mortality among young people^58^. Sixty young people died in L'Aquila city, comprising 29 males and 31 females. Their deaths occurred in only 15 locations, most of them the scenes of spontaneous collapse of multiple-occupancy apartment blocks. One of these was the student dormitory mentioned above, the *Casa dello Studente*(11 deaths; Figure 12). The tendency of students to live in modest, inexpensive accommodation proved fatal for some of them at L'Aquila.

**Zonation of mortality in relation to building collapse in L'Aquila city centre.**
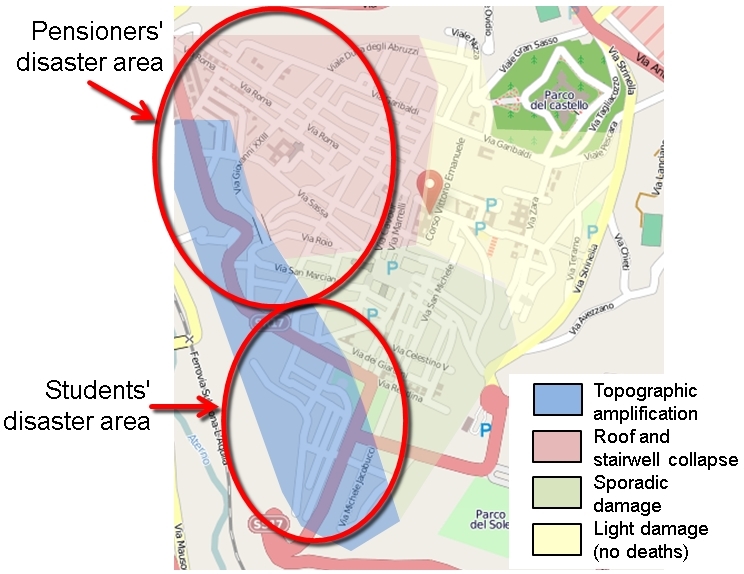
Image produced using maps from © OpenStreetMap contributors

**Collapsed student dormitory (Casa dello Studente), L'Aquila city.**
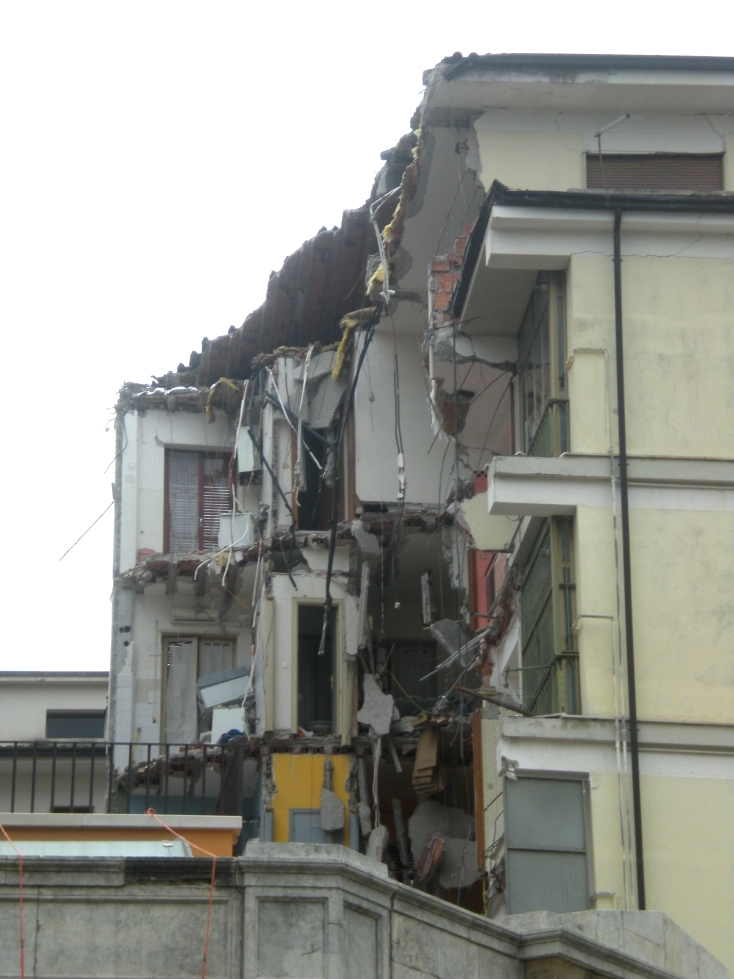

Finally, the literature points to a gender imbalance in seismic mortality statistics and this looms very large in the L'Aquila data set. The difference is very marked in old age, yet it cannot be explained by the fact that more elderly women than men are alive, as correcting for this fact does not remove the anomaly. Overall, the excess of women victims over men is about one third (177 men against 131 women). Women in L'Aquila Province constitute only 51.3% of the population, but they make up 57.5 per cent of the victims. In old age (70-96 years), the calculated excess of women over the demographic predictor is 40, or 13% of the total mortality. For men the figure is 19 excess deaths, or 6.2% of total mortality. Moreover, in the age range 30-39 the deaths of twice as many women as men (16 females and 8 males) is difficult to explain. Following Beinin (1981), one hypothesis was that women made more effort to save children. However, the death toll among small children was (thankfully) anomalously low. The behavioural information that we have is that there appears to have been no difference between male and female parents regarding their actions to save their offspring.

Detailed investigation of the deaths of 12 women and 4 men in the 30-39 age groups (and any survivors that were with them in their houses) in L'Aquila city centre revealed no indication of different causes of death, as spontaneous building collapse was overwhelmingly the culprit. The same was true for other age groups and locations. Extended interviews with survivors suggest the following two explanations. They both relate to the fact that the earthquake was nocturnal and that it was preceded three hours earlier, at 00.30 hrs, by a thoroughly alarming foreshock. People who left their homes after this were confronted with the choice of whether to remain outside, go to a safer location or return to bed. In the final analysis, the last of these options was the most dangerous, although local people could not have known that at the time. We noted a tendency among women interviewees to be more fatalistic than men and more disposed to treat the home as a refuge. Men were more likely to go away for the rest of the night or assume an attitude of readiness. Evidently, this was the safer course to take.

## Conclusion

Mortality patterns arising from the L'Aquila earthquake indicate a bias towards pensioners, students and women. More than twice as many pensioners died as the demographic profile would predict. By virtue of their agility and acute perception, young people should have suffered low mortality, but instead they dominate the statistics. The preponderance of female victims is evident in most age-groups, but especially in old age. As in all earthquakes, building collapse is the overwhelming main cause of death. Indeed, in L'Aquila, the spontaneous collapse of people's homes was almost the only cause of death. Evidently, these three groups were more at risk of perishing due to building collapse than other groups of people.

The exact causes of mortality in earthquakes remain controversial, because the literature is unsystematic, data sets may be incompatible and a wide variety of circumstances is revealed by studies of different events. Although the plight of the elderly has been recognised widely, that of young people and women has yet to be investigated properly. Failure to test for gender biases in many of the studies means that there the question of whether earthquakes discriminate against women has too often been culpably ignored. The evidence from L'Aquila is clear that women are more at risk from seismic disasters than are men. This fact needs to be taken into account in planning remedial measures and needs to be corrected through education programmes and special measures.

## Ethics Statement

All field surveys and analyses connected with the research described in this paper were carried out in accordance with policies adopted by the Polytechnic University of the Marche (UNIVPM), the second author's home institution. Living subjects were given a written questionnaire to fill in: it contained full information on the nature of the survey and the exact uses to which it would be put. They were informed in writing that by filling in the questionnaire they were thereby giving their consent to the uses of the information that they provided for the purposes explained in the selfsame document. They were, of course, given every opportunity not to participate if they did not agree with these uses. UNIVPM does not have a specific committee for the approval of research of this kind, as it is amply safeguarded, in extenso, by Italian national law DL 30-6-2003, no 196, "Codice in materia di protezione dei dati personali" (Code for the Protection of Personal Data). Following the law, subjects were made aware of this using the standard form of words for obtaining consent. All other data used were already in the public domain and have nevertheless been fully anonymised, as were all data on human subjects used in this study.

## Competing Interests

The authors have declared that no competing interests exist.
